# Transanal Resection of Rectal Lipoma Mimicking Rectal Prolapse: Description of a Case and Review of the Literature

**DOI:** 10.5402/2011/170285

**Published:** 2011-04-10

**Authors:** Jacopo Martellucci, Serenella Civitelli, Gabriello Tanzini

**Affiliations:** ^1^Department of General Surgery, University of Siena 53100 Siena, Italy; ^2^Chirurgia Generale I, Dipartimento di Chirurgia, Ospedale Policlinico Le Scotte, Viale Bracci 16, 53100 Siena, Italy

## Abstract

Submucosal lipomas of the large bowel are uncommon. Occasionally, they occur in the rectum and may cause aspecific symptoms; presentation with rectal prolapse is very unusual and may lead to a misdiagnosis of simple mucosal prolapse. The paper describes an additional case of a prolapsing rectal mass that led to diagnosis and surgical treatment of a rectal lipoma under local anesthesia.

## 1. Case Report

A 80-year-old man presented with a recent onset of constipation, anal discomfort, and anal incontinence.

Several months ago, he experienced a sensation of something passing through the anus at defecation, that he manually reinserted, accompanied by tenesmus and rectal bleeding, for which he had a diagnosis of “hemorrhoids”.

These symptoms worsened in the following months, since the mass began to prolapse independently of defecation, became painful when standing or walking, made defecation obstructed, and caused anal incontinence, for which he was referred with a presumptive diagnosis of neoplasia. 

Abdominal examination was unremarkable. 

Inspection of anal region revealed a large, soft, ulcerated mass protruding through the anus that was suspected for a submucosal neoplasia. The mass was easily reducible but prolapsed again.

At operation, the mass was pulled through the anal canal, and it was noted that it arises in the posterior wall of the rectum, 3 centimetres above the dentate line, where it developed a pseudostalk, presumably as a result of the repeated prolapses. The mucosa above the mass was injected with lidocaine and incised. The mass was sharply dissected and removed. The macroscopic appearance was that of a lipoma. The diameter was 9 centimetres. The mucosal edges were reapproximated by an absorbable running suture.

Histological examination confirmed the diagnosis of lipoma.

## 2. Discussion

Lipomas of the large intestine are relatively uncommon in clinical practice, although they are considered to be the benign tumours that occur most frequently, adenomas excluded, and the commonest nonepithelial tumours.

Most affected patients are in the 5th to 7th decades of life. Some authors have reported a female predominance while others found a nearly equal sex incidence [1]. In general, most lipomas are silent, and most series in the literature are autoptic reports in which their incidence ranges from 0.35 to 4.4% [2]. The number of large bowel lipomas found incidentally at necropsy far exceed the number of surgically treated cases [3]. The symptomatic lipomas are of clinical importance because of the many diagnostic and therapeutic problems they may cause. The major clinical problem is to differentiate lipomas from adenomas or polypoid cancer. Clinical signs are reported in a poor percentage of patients and are related to tumour location and size. 

Symptoms, that include bleeding, constipation, changing bowel habits, abdominal pain, intestinal obstruction, or intussusception of the mass [3–7], are more common when the lipoma is greater than 2 cm in diameter.

Spontaneous expulsion of a sigmoid lipoma has been reported [8].

Lesions are sessile or polypoid and are submucosal, in about 90% of cases, or subserosal.

In size, the tumours range from a few millimetres to several centimetres (0.5–8 cm in the serie of Castro and Stearns [9]; 4–21 in that of Michowitz et al. [10]; 0.5–10 in that of Rogy et al. [11]).

In some percentage, the lipomas are two or more (24.4%) [9] and not rarely associated with other pathologies, namely, colorectal cancer. Anyway, the high incidence of this association in some reports (33.3%) [9] does not reflect the actual incidence in the general population, but it reflects that of a cancer-patient population.

The most common site of origin is the right colon [3, 9]. There is no explanation for the predilection of lipomas of the large bowel to occur in the right side. Lipomas of the rectum are quite rare, and, in the various studies, are reported in a small number of patients. In the serie of Castro and Stearns [9], only 5 out of 45 lipomas of the large bowel (11.1%) were in the rectum and only one of these was palpable. The size of the lipomas of the rectum was recorded in 4 cases: 3 were less than 2 cm, and one was 2–4 cm in diameter.

Haller and Roberts reviewed 29 lipomas of the large bowel in 20 patients, none of which was in the rectum [2].

Michowitz et al., found only one lipoma of the rectosigmoid (4.54%) in his series of 22 cases [10].

In an 18-year study, Rogy et al. reported 17 patients with lipomas of the large bowel, and only three out of 21 were in the rectum [11].

Bozza et al. reported 11 patients with large bowel lipomas, all located proximal to the sigmoid colon [12]. 

The prolapse of a rectal lipoma through the anus is a rare event, and a few cases are reported in the literature [13, 14]. 

Taylor and Wolff, in a review of the Mayo clinic experience of 91 surgically managed colonic lipomas from 1976 to 1985, report no rectal location [15].

Ryan et al., in a review of the surgical pathology files, over a 50-year period, found 13 cases of colonic lipomas, none of these were in the rectum [16].

Creasy et al. identified 5 patients with symptomatic lipoma of the large bowel, searching the computerized histopathologic records of the Leicester General Hospital and Leicester Royal Infirmary, in a 11-year period, located, respectively in proximal colon (2), sigmoid colon (2), and rectum (1) [1].

**Figure 1:**
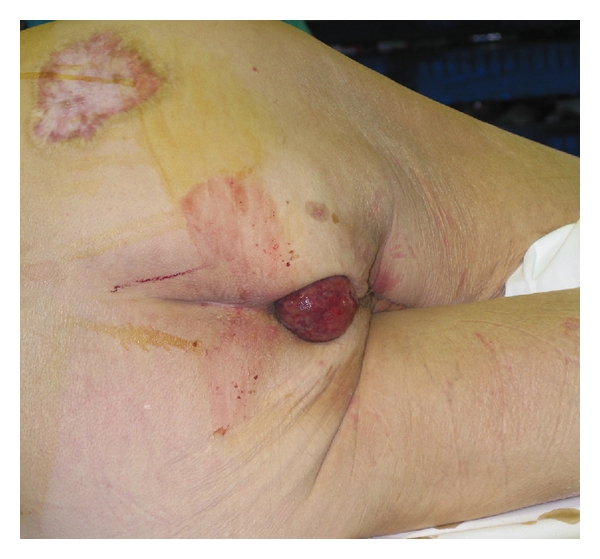
Lipoma of the rectum mimicking rectal prolapse.

**Figure 2:**
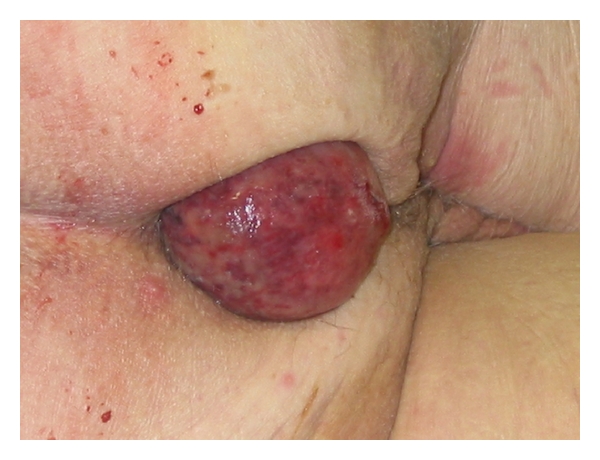
Lipoma in detail.

The patient with the rectal lipoma was a 35-year-old female presented as an emergency with lower abdominal pain and prolapsed rectal polyp, 6 centimetres in diameter (see Figures 1 and 2).

In a review of 11 patients treated for symptomatic lipomas of the gastrointestinal tract, during the period 1966–1972, Ackerman and Chughtai found the tumour located in the stomach (3 cases), in the ileocaecal region (4), and in the colon (3); the more distal lipoma was found in the sigmoid colon [17].
